# A promising new therapy may assist efforts to combat ICU-acquired weakness

**DOI:** 10.1186/s13054-014-0573-2

**Published:** 2014-10-23

**Authors:** Sarah Bain, Meagan Littlepage

**Affiliations:** Department of Anesthesia, El Camino Hospital, 2500 Grant Road, Mountain View, CA 94040, USA; Department of Physical Medicine and Rehabilitation, El Camino Hospital, 2500 Grant Road, Mountain View, CA 94040 USA

In previous issues of *Critical Care* and other publications, authors have characterized emerging therapies such as neuromuscular electrical stimulation (NMES) and functional electrical stimulation (FES)-cycling as promising tools to help prevent the onset of ICU-acquired weakness [1-3]. Despite compelling preliminary data, practical challenges with these technologies have limited their use. For example, there are documented limitations in NMES efficacy in patients with obesity and peripheral limb edema [3], and one study demonstrated treatment failures in 50% of patients, even in a non-obese cohort [4]. FES-cycling appears to perform more consistently (80% success) [2], but is associated with relatively long set-up times (15 to 30 minutes for a two-person team [2]) that make widespread use challenging when available resources are limited.

Motivated by recently presented research [5], we’ve deployed a newly available variation of NMES, thermal-enhanced muscle stimulation (supplied via the Niveus Medical System 110), in our critical care unit. This technique combines local hypothermia with electrical energy delivery in a manner that was shown to increase effectiveness and improve comfort [5]. While not intended to replace early team-driven mobilization, we hoped that this new method could provide more consistent efficacy than traditional NMES and facilitate our early mobility initiatives without adding significant burden to our staff.

Our experience led to several notable findings. Though our results are anecdotal, treated patients have consistently exceeded our expectations with regard to both strength and level of function following extended periods of immobilization. Additionally, we estimate that successful treatments have occurred in greater than 95% of sessions, which is substantially higher than reports of traditional NMES effectiveness in this cohort. To date, meaningful muscle contractions have been documented in patients with a body mass index as high as 42 and in patients with significant edema (for example, in a patient with a 21 kilogram weight gain from admission). With regard to patient comfort, it is common to see alert patients calmly experiencing quadriceps contractions strong enough to elevate heels from the bed bilaterally. Lastly, we found that bedside nursing can set up treatment in less than 5 minutes, minimizing distraction from other aspects of patient care and early rehabilitation.

Our experience using thermal-enhanced muscle stimulation has been overwhelmingly positive. We believe this technique is simple to deploy and could be a strong complement to other activities included in the ABCDE bundle. We hope our experiences will be informative to others when developing their own strategies for earlier rehabilitation in critically ill patients.

## Abbreviations

FES: Functional electrical stimulation
NMES: Neuromuscular electrical stimulation
